# Phosphoinositide 3-Kinase Dependent Inhibition as a Broad Basis for Opponent Coding in Mammalian Olfactory Receptor Neurons

**DOI:** 10.1371/journal.pone.0061553

**Published:** 2013-04-09

**Authors:** Kirill Ukhanov, Elizabeth A. Corey, Barry W. Ache

**Affiliations:** 1 Whitney Laboratory, Center for Smell and Taste, McKnight Brain Institute; University of Florida, Gainesville, Florida, United States of America; 2 Departments of Biology and Neuroscience, University of Florida, Gainesville, Florida, United States of America; Monell Chemical Senses Center, United States of America

## Abstract

Phosphoinositide 3-kinase (PI3K) signaling has been implicated in mediating inhibitory odorant input to mammalian olfactory receptor neurons (ORNs). To better understand the breadth of such inhibition in odor coding, we screened a panel of odorants representing different chemical classes, as well as odorants known to occur in a natural odor object (tomato), for their ability to rapidly activate PI3K-dependent inhibitory signaling. Odorants were screened on dissociated native rat ORNs before and after pre-incubation with the PI3K-isoform specific blockers AS252424 and TGX221. Many different odorants increased their excitatory strength for particular ORNs following PI3K blockade in a manner consistent with activating PI3K-dependent inhibitory signaling in those cells. The PI3K-dependent inhibitory odorants overlapped with conventional excitatory odorants, but did not share the same bias, indicating partial partitioning of the odor space. Finding that PI3K-dependent inhibition can be activated by a wide range of otherwise conventional excitatory odorants strongly implies PI3K-dependent inhibition provides a broad basis for opponent coding in mammalian ORNs.

## Introduction

Odor recognition is not just a simple summation of responses to the components of an odorant mixture. It has long been known to involve the synthesis of complex, poorly understood interactions variously described by terms like inhibition, suppression, hypoadditivity, masking, and synergy [1], [2], [3], [4], [5]. Odor recognition is also known to be a distributed phenomenon, although one that begins at the level of the olfactory receptor neuron (ORN), where different odorants generate complex patterns of activation ranging from strong activation to complete inhibition.

Evidence for inhibitory odorant interactions within complex mixtures has been found in many different species, arguing that inhibitory or opponent signaling is a fundamental principle of odor coding [1], [6], [7], [8]. Yet, the mechanisms by which odorants antagonize or oppose one another are not well understood. In mammals, antagonists structurally related to known agonists, e.g., methyl isoeugenol *versus* eugenol, can directly interfere with ORN activation in a manner indicative of, or at least strongly suggestive of, competitive inhibition at the binding pocket of the olfactory receptor (OR) [9], [10], [11], [12], [13]. In contrast, chemically dissimilar odorants that vary in agonistic strength, e.g., citral *versus* octanol, can inhibit mammalian ORNs in a phosphoinositide 3-kinase (PI3K)-dependent manner [14], [15], suggesting a different mechanism of antagonism or opposition in which odorants rapidly activate an opponent signaling pathway.

Understanding the overall contribution and mechanistic nature of inhibitory input to olfactory coding first requires addressing some basic questions that have largely gone unanswered. Are there dedicated ‘inhibitory’ odorants and how many are there? Can ‘inhibitory’ odorants be assigned to a particular subset of odorants? Is there relationship between the inhibitory and conventional excitatory odorants for a given OR? Here we address these questions in the context of PI3K-dependent inhibition. We show that there is a large repertoire of odorants from diverse molecular classes that can activate PI3K in a manner physiologically relevant to olfactory transduction, including those occurring in a behaviorally-salient natural odor mixture. We further show that the PI3K-dependent inhibitory odorants overlap with conventional excitatory odorants, but do not share the same bias, indicating partial partitioning of the odor space. Finding that PI3K-dependent inhibition can be activated by a wide range of otherwise conventional excitatory odorants strongly implies PI3K-dependent inhibition provides a broad basis for opponent coding in mammalian ORNs.

## Materials and Methods

All experiments were performed on adult female Sprague-Dawley rats. All procedures were carried out in accordance with protocols approved by the University of Florida IACUC, protocol # 201105796. Rats were euthanized by inhalation of carbon dioxide and decapitated. All experiments were performed at room temperature (22–25°C).

### Calcium imaging

Experiments were performed as described previously [14], [15]. In brief, olfactory tissue was dissected in ice-cold modified artificial cerebrospinal fluid (ACSF) saturated with 95% O_2_ and 5% CO_2_ that contained (in mM): 120 NaCl, 25 NaHCO_3_, 5 KCl, 1.25 Na_2_HPO_4_, 1 MgSO_4_, 1 CaCl_2_, 10 glucose, 305 mOsm, pH7.4. After enzymatic digestion at 37°C, the tissue was gently washed with normal oxygenated ACSF and accurately triturated with a large bore fire polished glass transfer pipette. The resulting suspension was filtered through a 40 µm cell strainer (Fisher Scientific) and stored at 4°C until needed. An aliquot of the suspension was mixed with 10 µM Fluo-3/AM (AnaSpec) containing 0.04% Pluronic F127 and placed on a glass coverslip coated with concanavalin A (Sigma-Aldrich) in a recording chamber (RC22, Warner Instruments). The chamber was transferred to the stage of an inverted microscope (Axiovert 200, Zeiss) equipped with a 10×/0.5NA Fluar objective. To increase the sample size in each experimental session the field of view was further expanded using a 0.63× reducer video tube (Diagnostic Instruments) between the microscope and the camera. Depending on the quality of the preparation, 300–600 ORNs sensitive to IBMX/forskolin stimulation could be analyzed at once. The cells were illuminated at 500 nm (BP 500/20 nm, Omega Optical, USA) and the emitted light was collected at 530 nm (BP 530/20 nm, Omega Optical, USA) by a 12-bit cooled CCD camera (ORCA R2, Hamamatsu, Japan). Both the illumination system (Lambda DG-4, Sutter Instruments, USA) and image acquisition were controlled by Imaging Workbench 6 software (INDEC BioSystems).

Each cell was assigned a region of interest (ROI) and changes in fluorescence intensity within each ROI were analyzed and expressed as the peak fractional change in fluorescent light intensity F/F_0_ where F_0_ is the baseline fluorescence before odorant application. Response was detected and measured when the change in fluorescence intensity exceeded two standard deviations above the noise median level. For quantitative comparison, the peak amplitudes of the responses of different cells were normalized to the saturated responses elicited by application of a mixture of 100 µM IBMX (a phosphodiesterase inhibitor) and 10 µM forskolin (a selective agonist of adenylate cyclases) to robustly activate the cyclic nucleotide signaling pathway. Application of IBMX/forskolin was also used to identify functional ORNs. We elected to use this stimulation instead of applying saline with high KCl since often acutely dissociated cells lack dendrites and cilia yet are still able to react to the membrane depolarization induced by high KCl. The resulting values are referred to as relative units. In each experiment, groups of nine odorants were tested and then the results of all experiments representing survey of 42 odorants were pooled for further analysis. Special attention was paid to assure we were imaging individual cells when several ORNs in a cluster were co-activated.

### Reagents and solution application

3-isobutyl-1-methylxanthine (IBMX) and 7β-acetoxy-8,13-epoxy-1α,6β,9α-trihydroxylabd-14-en-11-one (forskolin) were from Sigma-Aldrich. Odorants were delivered as aqueous solutions prepared in freshly oxygenated ACSF. Single odorants were of highest purity obtained from Sigma-Aldrich, Acros Chemicals or Alpha Aesar (purchased through Fisher Scientific) and were prepared as 0.5 M stock solution in anhydrous DMSO. Cycloheptanecarbaldehyde was purchased from Wako Pure Chemical Industries (Japan). All odorant stocks were kept frozen at -20°C and the final aqueous solutions were prepared on the day of experiment. ACSF supplemented with 0.1% DMSO, the odorant carrier, served as the control solution. PI3K beta and gamma isoform-specific blockers TGX221 and AS252525 (Cayman Chemical) were supplied as 3.3 mM solution in pure ethanol.

### Data analysis

All data are expressed as mean±SEM. Statistical and clustering analyses were performed using build-in functions in SigmaPlot 10 (Systat Software, USA) or XLSTAT 12 (Addinsoft, USA).

## Results

### Broad screen to identify PI3K-dependent inhibitory odorants

**Figure 1:**
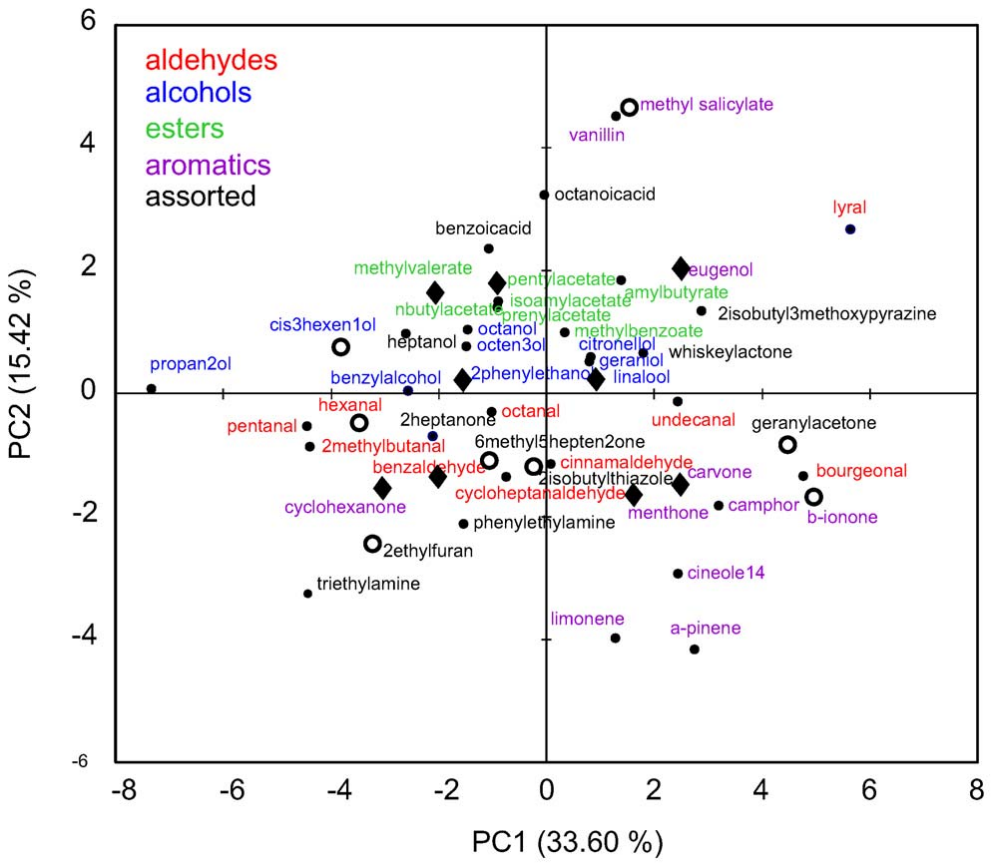
Physico-chemical representation of odorants used in the study. A set of 38 physico-chemical descriptors was generated using CHEMMINE Tools. Odorants are color coded according to their main chemical functional groups. The group of assorted odorants includes aliphatic acids, ketones, heterocyclic compounds and lactones. Principal component analysis (PCA) of all odorants used in the study is based on their physico-chemical descriptors. The first two principal components are shown; values in parentheses refer to the proportion of the observed variance in the data that was explained by PC1 and PC2. 42 odorants labeled with dots including a subset of 9 odorants (black diamonds) used to perform a focused analysis of the ORN response profiles. Additional 8 naturally occurring major components of the tomato odor (open circles) were also included in the dataset.

To address the question of whether there are dedicated PI3K-dependent inhibitory odorants, we began by assembling a panel of 42 conventional odorants, all of which are known to elicit olfactory responses in rodents. These odorants represented a relatively broad range of chemical features, such as carbon chain length and functional group, with sufficient diversity to approximate the odor space of rats. Odorants were analyzed using an optimized set of 38 chemical descriptors generated by CHEMMINE Tools [16]. Included in this group were odorants that also fall into the distinct subsets as defined by Haddad et al. [17] and classes as defined by Bachtiar et al. [18]. To reduce the dimensionality of the dataset, we used principal component analysis (PCA) to represent each odorant by two parameters such that odorants were no longer defined by their pair-wise similarity to all the other odorants (9 dimensions) but rather by the first two principal components (PC1, PC2 – Figure 1). PCA analysis reveals a rather sparse distribution of the odorants in physico-chemical space, suggesting a broad representation of chemical features. Based on the results of these analyses, we grouped the odorants into five chemical classes including aldehydes, alcohols, esters, aromatics and ‘assorted’ (aliphatic acids, ketones, heterocyclic compounds, and lactones).

**Figure 2:**
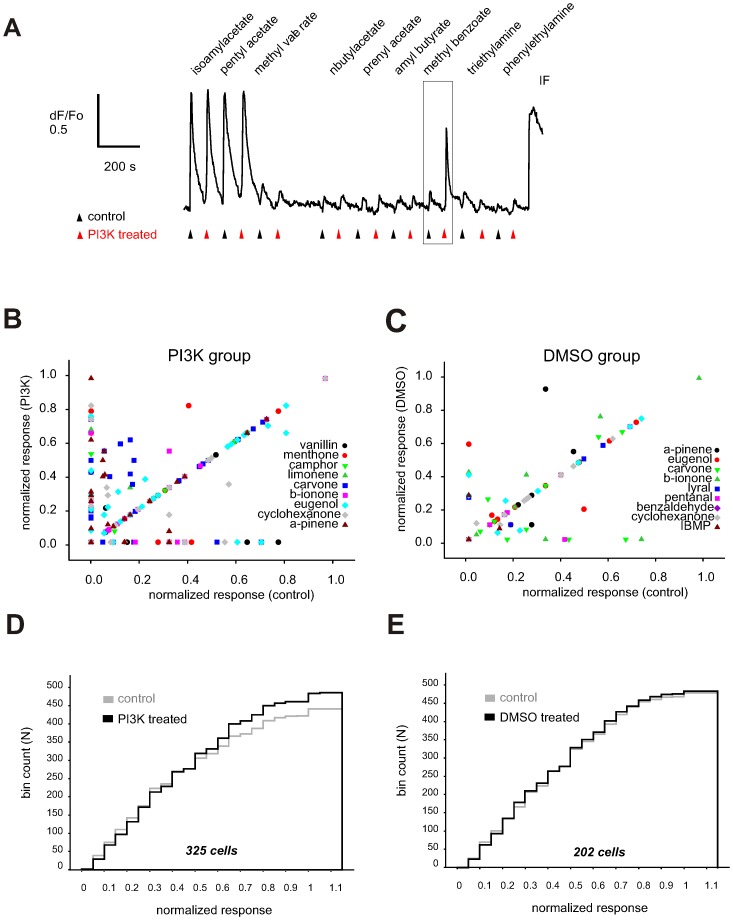
Screening dissociated rat ORNs for PI3K-dependent inhibitory odorants. (A) A representative recording of the fluo-3 calcium responses evoked by a 10-s pulse of indicated odorant (each at 50 µM) (control, black triangles) followed by a second pulse after 15 s pre-incubation with TGX221 and AS252424 (each at 200 nM), the PI3K ß and γ isoform selective inhibitors (PI3K-treated, red triangles). Each experimental trial involved 9 different odorants and was completed by application of IBMX/forskolin (100/10 µM, IF) inducing a saturated calcium elevation used for the response normalization. (B,C) A representative graph of the normalized responses induced by 9 odorants in PI3K-inhibitor treated group (B) and in DMSO-treated group of cells (C) as assayed in two independent experiments. Normalized response amplitude following either PI3K blockade or pre-incubation with 0.1% DMSO was plotted against that in the control. Odorants coded with different symbols shown in different colors. Number of cells in each group is shown at the bottom. (D) Cumulative histogram of normalized response amplitude of the odorant responses to 42 odorants recorded from a total of 325 cells representing the PI3K inhibitor-treated group. The PI3K-dependent inhibition is evident as a shift towards larger amplitudes values when comparing the control (gray line) and PI3K inhibitor-treated group (black line). (E) A similar cumulative histogram of normalized responses to the same odorants recorded from a different 202 cells representing the DMSO-treated group. Both data sets yield essentially identical histogram in the control (gray line) and DMSO-treated group (black line). Odorant name 2-isobutyl-3-methoxypyrazine is abbreviated as IBMP. Odorant names and symbols are color coded.

We screened the odorants at a single concentration (50 µM) on a large group of rat ORNs sensitive to IBMX/forskolin stimulation (IF+ ORNs) for the ability to evoke PI3K-dependent inhibition (Fig. 2). The calcium signal evoked by each odorant was measured before and after pre-treatment with PI3K-specific blockers (TGX221 and AS252424, 200 nM each) and any change in the amplitude of the signal following pharmacological blockade noted. Fig. 2A shows a representative recording of a cell in which isoamyl acetate and pentyl acetate were strongly excitatory before PI3K blockade that changed little after blockade. In contrast, methyl benzoate was only weakly excitatory for the cell before PI3K blockade and the response significantly increased after blockade (Fig. 2A boxed area). Here, and throughout the paper we refer to the responses to isoamyl acetate and pentyl acetate as conventional excitation and that to methyl benzoate as PI3K-dependent inhibition, and to isoamyl acetate and pentyl acetate as conventional excitatory odorants and to methyl benzoate as a PI3K-dependent inhibitory odorant *for that cell*.

Of 7,664 acutely dissociated rat IF+ ORNs 325 responded to at least one of the 42 individual odorants before and/or after PI3K blockade (Fig. 2). To determine whether PI3K activation consistently resulted in inhibition, responses were normalized to the saturating response elicited by application of IBMX/forskolin (100/10 µM) and then the results were plotted against the normalized response for all cells tested (Fig. 2B). PI3K-blockade increased the amplitude of the response in 229 out of 2,970 (7.7%) cell-odorant combinations represented by the data points above the diagonal line in Fig. 2B. Analysis of the distribution of all of the responses before and after PI3K blockade plotted as cumulative histograms (Fig. 2D) shows that PI3K blockade resulted in a significantly larger amplitude (mean normalized amplitude 0.27±0.07 (control, 1^st^ response) vs 0.36±0.07 (PI3K-treated, 2^nd^ response) (Z = 4.442, p<0.001, n = 2970, Wilcoxon signed rank test). In a number of cases (3.6%), PI3K blockade decreased the amplitude of the response (points below the diagonal in Fig. 2B). This was likely the result of desensitization and/or random variability since in 202 ORNs (yielding 2169 cell-odorant combinations) treated with DMSO instead of PI3K blockers, the response to the second application of the same odorant was reduced in 2.7% of the cell-odorant combinations and increased in 3.1%. The resulting cumulative amplitude histograms were overlapping (Fig. 2E) under both experimental conditions (mean normalized amplitude 0.369±0.051 before DMSO treatment vs 0.374±0.049 after DMSO treatment) (Z = 0.141, p = 0.888, n = 2169, Wilcoxon signed rank test), indicating that in contrast to PI3K blockade, DMSO treatment did not significantly affect the amplitude of the odorant responses. Overall, these results demonstrate that most of the odorants tested were able to evoke PI3K-dependent inhibition in at least some ORNs, so there do not appear to be any dedicated ‘inhibitory’ odorants.

### Incidence of PI3K-dependent inhibitory odorants

**Figure 3:**
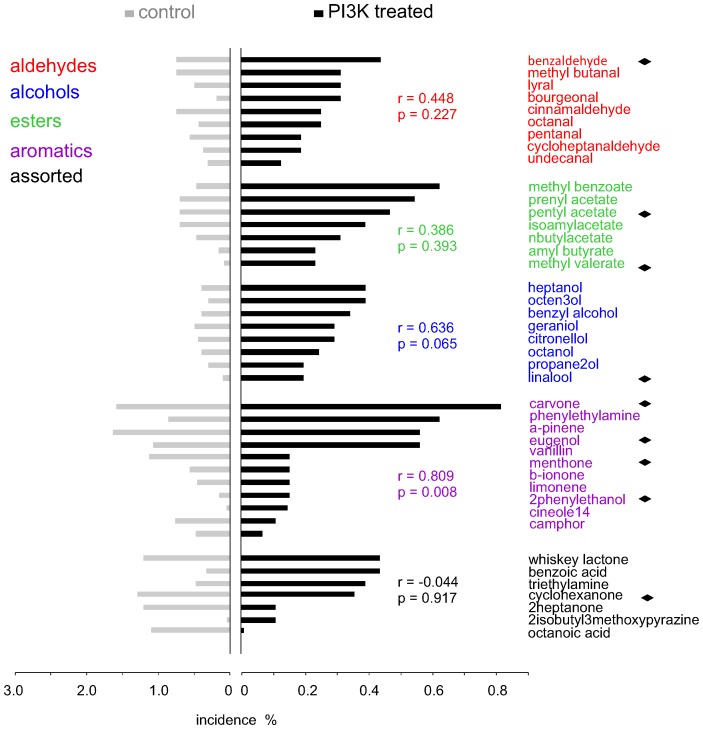
Summary of the response incidence of each of 42 odorants applied at 50 µM before (gray) and after PI3K blockade (black bar) which is expressed as a fraction of cells responsive to IBMX/forskolin and sensitive to at least one odorant tested. Incidence of the PI3K-dependent inhibitory odorants was calculated by counting only those responses that were *increased* after PI3K blockade. A subset of 9 chemical compounds (diamonds) was selected among 42 odorants for a more detailed analysis of PI3K-dependent inhibition. A correlation between the incidence before and after PI3K blockade (PI3K treated) within each chemical class was analyzed using Pearson test, which failed to reveal a significant dependency except for one group of aromatic odorants. Pearson correlation coefficient (r) and probability value (p) is shown for each group of odorants. Odorant names and symbols are color coded according to the main chemical functional groups.

To address the question of whether any odorant class evoked PI3K-dependent inhibition more often than others, we plotted the incidence of the responses (the percentage of the total IF+ ORNs activated) to each odorant before and after PI3K blockade against the chemical class of the odorant. The odorants are sparsely distributed along the incidence scale (Fig. 3) and there is no clear relationship between chemical class and PI3K-dependent inhibition. These results underscore that many different odorants are capable of activating PI3K-dependent inhibition and indicate that they cannot be assigned to a specific chemical class.

While there was no particular class that was responsible for evoking PI3K-dependent inhibition, odorants within chemical classes with few chemical features (e.g., alcohols, aldehydes, and esters, Fig. 1) had less variability in the incidence of PI3K-dependency than odorants in classes displaying more chemical features (e.g., aromatics and assorted groups, Fig. 1) (Fig. 3). For example, the incidence of PI3K-dependent inhibitory responses within the alcohols ranged from 0.2 to 0.4%, while within the aromatics it ranged from 0.07 to 0.83%. However, this greater level of variability in response to the aromatic and assorted odorant classes is also seen in the conventional excitatory responses and presumably is not unique to PI3K-dependent odorants (Fig. 3).

As demonstrated for excitatory odorants in mammalian ORNs [19], [20], [21], some odorants activated PI3K-dependent inhibition in more ORNs than did others. i.e., there is bias in the receptor population for odorants evoking PI3K-dependent inhibition. The incidence of PI3K-dependent inhibition ranged from a low of 0.0, 0.07 and 0.11%, respectively, for octanoic acid, camphor and 2-isobutyl-3-methoxypyrazine to 0.63, 0.63 and 0.83%, respectively, for methyl benzoate, phenyl ethylamine and carvone. For comparison, considering the incidence of conventional excitatory responses prior to PI3K blockade, 2-isobutyl-3-methoxypyrazine, 2-phenyl ethanol and methyl valerate had the lowest incidence (0, 0.05 and 0.08%, respectively), while cyclohexanone, carvone, and eugenol had the highest (1.31; 1.61 and 1.66%, respectively). This would suggest that the bias in the receptor population for odorants evoking PI3K-dependent inhibition does not align with the bias of the same odorants to evoke conventional excitatory responses in the same sample population. That idea is supported by the absence of correlation between the incidence of the response to odorants before and following PI3K blockade for each chemical class of odorant tested (Fig. 3, correlation coefficients shown for each class). Only the aromatic compounds showed significant (r = 0.81, p = 0.01) positive association between the incidence of the response to odorants before and following PI3K blockade, although this could potentially reflect more extreme variability in these data (Fig. 3, aromatics).

### Relationship between PI3K-dependent inhibitory and conventional excitatory odorants on individual ORNs

**Figure 4:**
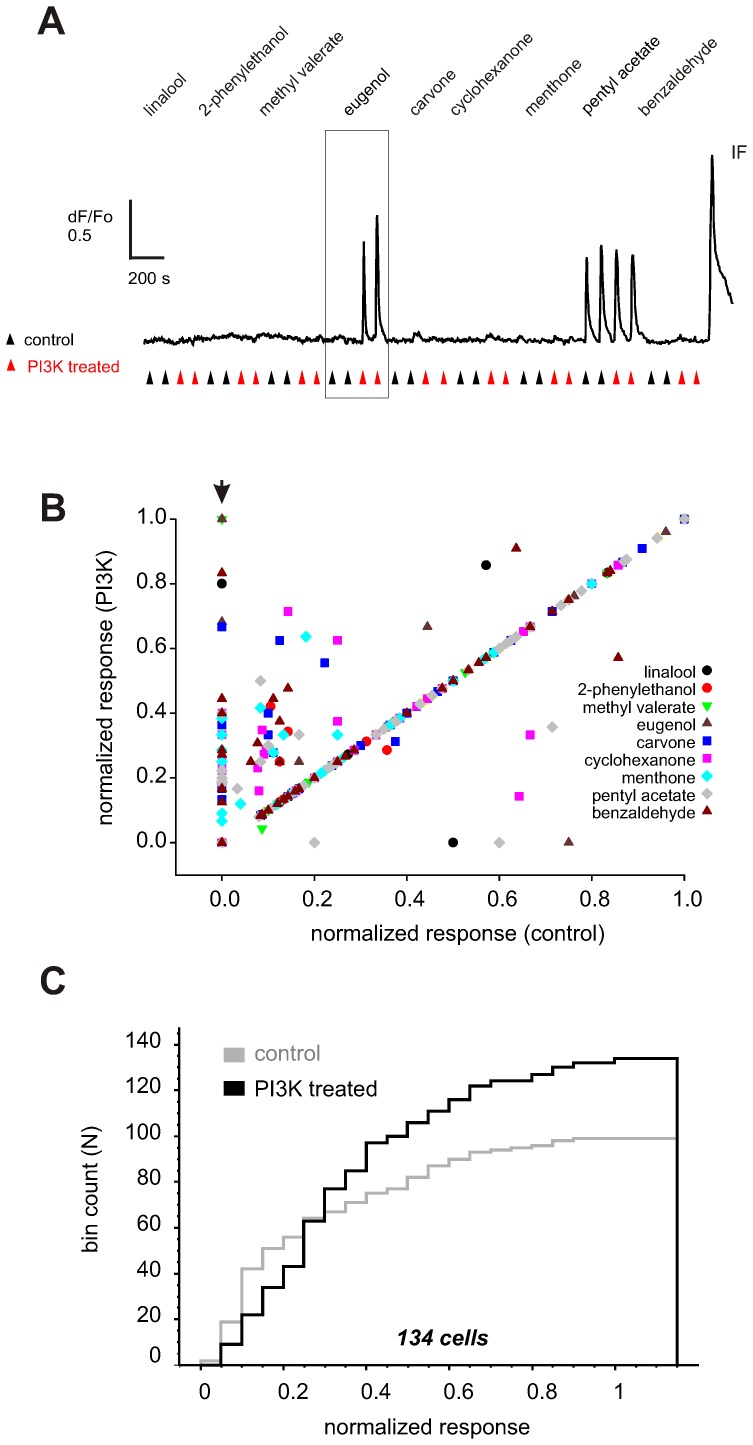
A more stringent analysis of PI3K-dependent inhibitory responses evoked by 9 odorants (diamonds inFigure 3) applied on a different set of 134 cells. (A) Two pulses of 50 µM odorant were applied before (control, gray triangles) and after PI3K blockade (black triangles). A representative recording from a dissociated ORN sensitive to conventional excitatory odorant pentyl acetate whereby eugenol performed as a PI3K-dependent inhibitory odorant on this cell (boxed area). A total of 51 cells were identified in such a manner. A saturated IBMX/forskolin response (IF) was used to normalize the response evoked by each odorant. (B) Average amplitude was calculated for each pair of responses and normalized to the saturated IBMX/forskolin response. Normalized response amplitude after PI3K blockade was plotted against that before blockade. Many cells that were not at all or only weakly activated prior to PI3K blockade responded more strongly afterwards (arrowhead). Odorants coded with different symbols shown in different colors. Number of cells in the group is shown at the bottom. (C) Cumulative response amplitude histogram of the odorant responses plotted in B. Pre-incubation with the PI3K inhibitors (black line) significantly shifts the histogram to a larger values compared to before treatment (gray line).

To understand the relationship between odorants evoking PI3K-dependent inhibitory and conventional excitatory responses on individual ORNs, we performed a more stringent paired pulse screen of a different population of 2,840 IF+ ORNs using a subset of nine odorants selected from the low, middle, and high parts of the incidence range (Fig. 3, diamonds). To better control for potential false positives, every cell in this experiment was stimulated twice with 50 µM of each odorant before and after PI3K blockade (Fig. 4A). In the example shown pentyl acetate again performed as a conventional excitatory odorant whereby eugenol was a PI3K-dependent inhibitory odorant (Fig. 4A). 134 of the ORNs responded to at least one of the nine odorants tested either before and/or after PI3K blockade, and 51 of those cells (38%) showed PI3K-dependent inhibitory responses to at least one odorant. As in the initial assay, PI3K blockade (Fig. 4B) almost always (6.1% of 1188 cell-odorant combinations) resulted in a significant increase in response amplitude. In comparison a reduced response was observed following the PI3K blockade only in 0.9% cases (Fig. 4B). Comparison of the cumulative response amplitude histogram before and after PI3K blockade and the group average values [0.13±0.01 (control) vs 0.27±0.02 (PI3K treated group); Z = 5.46, p<0.001, n = 1188; Wilcoxon signed rank test] (Fig. 4C), indicates that the single pulse protocol in the initial broad screen did not impose significant error.

**Figure 5:**
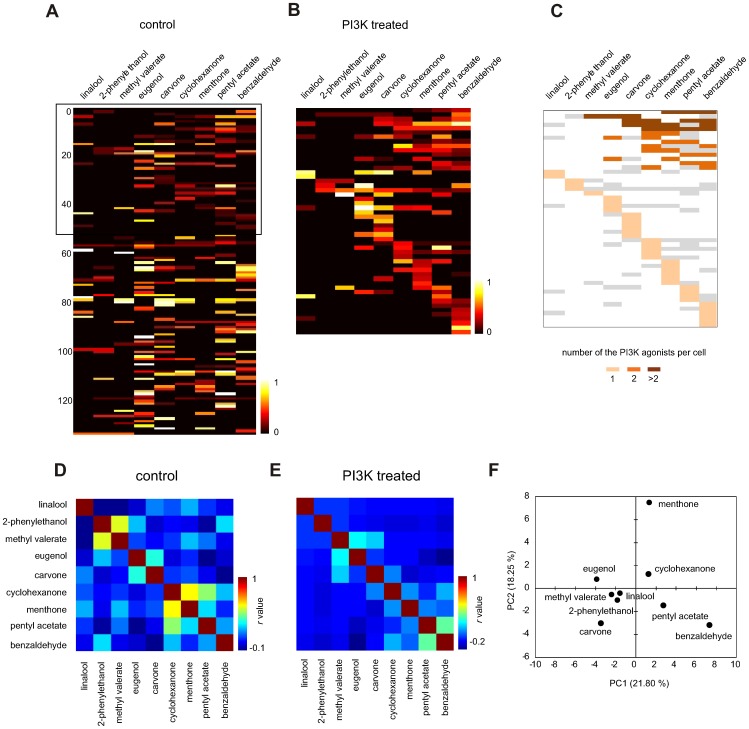
Odorant profiles of ORNs that responded to PI3K-dependent inhibitory odorants. (A) The data in Figure 4B is presented as a heatmap of responses from all 134 individual cells activated by at least a single of 9 odorants before PI3K blockade. 51 cells activated by the PI3K-dependent inhibitory odorants were grouped together in the top part (boxed area). (B) Heatmap of responses measured in the same 51 cells after PI3K blockade. Color scale is calibrated to display normalized response amplitude. (C) Cells were mapped by the PI3K-dependent inhibitory response and color-coded according to the number of PI3K-dependent inhibitory odorants per cell. Note that even cells that responded to multiple odorants only responded to a single odorant in a PI3K-dependent inhibitory manner. Gray blocks represent responses which remained unchanged whereby white blocks refer to zero response after PI3K blockade. (D, E) Analysis of the pair-wise similarity between each of 9 odorants tested. Neural response similarity plotted as an odorant correlation matrix before (D, control) and after blockade with PI3K inhibitors (E, PI3K treated). Values on the scale refer to the Pearson' correlation. Cross-correlation of the two matrices using a Mantel's test was insignificant (r(AB) -0.192, p-value (two-tailed) 0.234). (F) Mapping of odorants to neural space using principal component analysis (PCA) of the tested odorants based on the response similarity. The first two principal components are shown; values in parentheses denote the amount of the observed variance in the data that was explained by PC1 and PC2. Overall odorants were sparsely represented in the neural space.

In order to understand if there is any difference in the tuning of individual ORNs to PI3K-dependent inhibitory odorants compared to conventional excitatory odorants, the responses before and after PI3K blockade were normalized to the saturating response elicited by IBMX/forskolin and used to create heat maps (Fig. 5A,B). As previously demonstrated [19], [22], [23], mapping the conventional excitatory responses of all 134 odorant sensitive cells before PI3K blockade revealed various degrees of tuning, with 72% (96/134) responding to two or less odorants and 28% (38/134) to three or more. When just the cells that showed PI3K-dependent inhibitory responses were analyzed (Figure 5A, boxed area), 20% (10/51) failed to show a conventional excitatory response to any odorant before blockade, 24% (12/51) responded to a single odorant, and 57% (29/51) responded to multiple odorants. In comparison, the heat map of the responses after PI3K blockade (Fig. 5B) showed that the treatment increased the response to single odorants in 73% (37/51) ORNs and to multiple odorants in 27% (14/51) ORNs. Cluster analyses of the conventional excitatory responses with a 134×9 binary matrix revealed 56 distinct response profiles (data not shown), of which 31 were from the 51 cells that later showed PI3K-dependent inhibitory responses. In contrast, analysis of the PI3K-dependent inhibitory responses with a unitary 51×9 response map (Fig. 5C) revealed just 20 response profiles. This analysis suggests that individual ORNs are tuned to fewer PI3K-dependent inhibitory odorants than conventional excitatory odorants.

We then explored whether there was any correlation between the particular odorants that evoked PI3K-dependent inhibitory and conventional excitatory responses in the same ORN. To do this we calculated the pair-wise correlation matrix of the responses to the same nine odorants before and after PI3K blockade for the 51 cells that showed PI3K-dependent inhibition, giving one correlation coefficient per odorant pair (Fig. 5D,E). There was little association between the responses before and after PI3K blockade, presumably reflecting the significantly different physico-chemical properties of the odorants evoking the responses (Fig. 1 black diamonds). More importantly, comparison of the response profiles before and after PI3K blockade (Fig. 5D,E) revealed little cross-correlation [Mantel test, r(AB) -0.192, p-value (two-tailed) 0.234], suggesting that for a given odorant the two response types likely came from different ORNs. Using PCA analysis to reduce the dimensions of the data set and plotting the first two principal components, PC1 and PC2, for each odorant for the group of cells that showed PI3K-dependent inhibitory responses (Fig. 5F) indicates similar neural representation but very little association. These results suggest that for any given ORN, the odorant(s) that evoked PI3K-dependent inhibitory responses were not the same as the odorant(s) evoking conventional excitatory responses.

### PI3K-dependent inhibitory odorants in a natural odor source

To better understand the potential impact of PI3K-dependent inhibitory odorants on coding we examined volatiles found in a natural source. As some of the conventional odorants tested, including eugenol, ß-ionone, linalool, phenyl ethanol, benzaldehyde and pentyl acetate, occur in the volatile spectrum of ripe tomatoes [24], [25], [26], we used tomato as an ‘odor object’. Among the major tomato volatiles, we selected eight compounds that are metabolic derivatives downstream of fatty acids (hexanal, 2-ethylfuran, cis-3-hexen-1-ol), carotenoids (6-methyl-5-hepten-2-one, ß-ionone, geranyl acetone) and amino acids (2-isobutylthiazole, methyl salicylate) [24].

**Figure 6:**
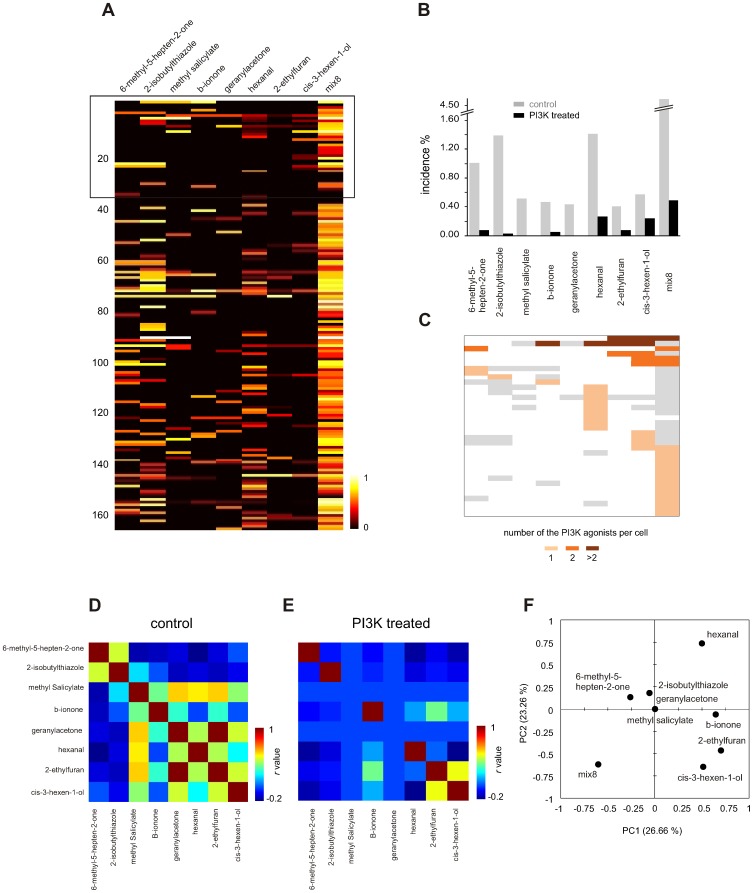
Major components of naturally occurring tomato odor also perform as a broad PI3K-dependent inhibitory odorants. (A) A heatmap of responses evoked by each of the 8 tomato odorants applied at 50 µM and their equimolar mixture (mix8) measured in a different 162 ORNs before PI3K blockade. 32 cells activated after PI3K blockade were grouped together in the top part (boxed area). (B) Summary of the response incidence of each odorant in before (gray) and after PI3K blockade (black bar, PI3K treated) which is expressed as a fraction of cells responsive to IBMX/forskolin and sensitive to at least one of the 8 odorants tested. Incidence of the PI3K-dependent inhibitory odorants was calculated by counting only those responses that were *increased* after PI3K blockade. (C) A total of 32 cells that responded to an odorant in PI3K-dependent inhibitory manner were grouped by the odorant and color-coded according to the number of the PI3K-dependent inhibitory odorants per cell. Gray blocks represent responses which remained unchanged whereby white blocks refer to zero response after PI3K blockade. (D,E) Analysis of the pair-wise similarity between each of the 8 odorants tested. Neural response similarity plotted as an odorant correlation matrix before (D, control) and after PI3K blockade (E, PI3K treated). Values on the scale refer to the Pearson's correlation. Cross-correlation of the two matrices using a Mantel's test was insignificant (r(AB) 0.381, p-value 0.093). (F) Mapping of odorants to the neural space using principal component analysis (PCA) of the tested odorants based on response similarity. As in the case of 9 random odorants (Fig.5F) the PI3K-dependent inhibitory odorants also were sparsely represented in the neural space.

An equimolar mixture of the eight (mix8), as well as each individual odorant (at 50 µM), was applied to a different population of 3,680 IF+ ORNs, using the protocol from the paired-pulse screen. The ORNs showed strong conventional excitatory and PI3K-dependent inhibitory responses to mix8 (Fig. 6A,B). 162 (4.4%) of the ORNs responded before and/or after PI3K blockade to at least one of the eight individual odorants. The three odorants with the highest incidence of response before blockade were hexanal, 2-isobutylthiazole and 6-methyl-5-hepten-2-one (1.4%, 1.4% and 1.0%, respectively), and many of the cells were broadly tuned (Fig. 6A). In comparison, hexanal, cis-3-hexen-1-ol and 6-methyl-5-hepten-2-one (0.27%, 0.24% and 0.08%, respectively), had the highest incidence of PI3K-dependent inhibitory responses. PI3K blockade increased the amplitude of the response in 36 of the 162 responsive cells (22.2%) to six of the odorants (Fig. 6B), suggesting that no single odorant in the mixture was responsible for PI3K-dependent inhibition.

We next examined the relationship between the conventional excitatory and PI3K-dependent inhibitory responses to the tomato volatiles. As with the 9 random odorants, ORN tuning to PI3K-dependent inhibitory odorants was narrower than to conventional excitatory odorants, and each odorant acted on a different type of ORN. Cluster analysis of the tomato odorant-evoked responses of just the 36 cells that showed PI3K-dependent inhibition reveals 15 conventional excitatory response profiles in comparison to just 10 PI3K-dependent inhibitory profiles. When odorants were correlated with the neuronal activity to build similarity matrices, comparison did not reveal a significant cross-correlation between activation patterns before and after PI3K blockade (Mantel test, r(AB) 0.381, p-value (two-tailed) 0.093). Additionally, PCA analysis of the data set shows that the PI3K-dependent inhibitory tomato odorants are sparsely distributed in the neural space, suggesting that each acts on a distinct ORN type (Fig. 6F). Overall, these results indicate that many naturally-occurring odorants found together in an odor-object activate PI3K-dependent inhibitory signaling.

## Discussion

Previously, we demonstrated that activation of rat ORNs could be inhibited in a PI3K-dependent manner by studying a few inhibitory odorant pairs in detail [14], [15]. We found that the agonist strength of the weaker, otherwise inhibitory, agonist of the pair was increased following PI3K blockade, suggesting the inhibition normally was mediated through a PI3K-dependent pathway. Using that same rationale, we can now report that such PI3K-dependent inhibition is not a selective property of the few odorant pairs tested, but rather a property inherent in a broad range of odorants representing different molecular classes, odorants that we refer to herein as PI3K-dependent inhibitory odorants. We base this conclusion on our ability to show that blocking PI3K significantly changed (increased) the response of at least some ORNs to all of the selected ‘off the shelf’ odorants tested. Our ability to also show that blocking PI3K significantly changed (increased) the response of at least some ORNs to a number of the major volatiles found in tomato, a natural odor source, establishes the biological significance of the finding. Tomato volatiles are likely to be biologically salient to rodents, given rodents are natural dispersants of tomato seeds in the native habitat of the tomato [24], [27], [28].

It is important to have implicated PI3K signaling in a physiological context relative to olfactory transduction. PI3K signaling regulates multiple cellular processes such as proliferation, apoptosis, and intracellular trafficking [29], [30], including the survival of mammalian ORNs [31]. Since odorant stimulation enhances the survival of ORNs [32], it could be argued, for instance, that all odorants could activate PI3K in the context of preventing apoptosis. While that may be true, here we measured the effect of pharmacologically blocking the enzyme on the odorant-evoked calcium response of the cell. The odorant-evoked calcium response is still slow relative to transduction per se, but earlier we showed that the effect of pharmacologically blocking PI3K on the calcium response correlates with changing the slope of the onset of the excitatory electrophysiological response of the ORN [15]. The exact mechanism by which odorant-activated PI3K inhibits the excitatory response of the ORN remains unknown, but phosphatidylinositol (3,4,5)-trisphosphate, PIP_3_, the product of PI3K activation, negatively regulates the olfactory cyclic nucleotide-gated (CNG) channel [33], [34], suggesting that the CNG channel may be at least a downstream target of PI3K-mediated inhibition.

Interestingly, the incidence or bias of the PI3K-dependent inhibitory odorants did not correlate strongly with that of odorants shown in the present study and elsewhere to be conventional excitatory odorants. For example, the incidence of the responses to aliphatic aldehydes, alcohols, and acids, among the most potent excitatory odorants for rodents [19], [20], [35], before and following PI3K blockade failed to correlate (Fig. 3). Only among the aromatic odorants was the incidence of the response following PI3K blockade positively correlated with that prior to blockade (Fig. 3), and this may have reflected the extreme variability of these data. Similarly among the tomato volatiles, while hexanal had a high incidence of acting as both a strong excitatory odorant and a PI3K-dependent inhibitory odorant, other odorants that had an even higher incidence of being strong excitatory odorants had a low incidence of evoking PI3K-dependent inhibition (Fig. 6B). Thus, while the ability of a broad range of odorants to evoke PI3K-dependent responses, and the ability of an odorant to excite one ORN and inhibit another in a PI3K-dependent manner, indicate there is no clear functional subset of PI3K-dependent inhibitory odorants, the odor space appears to be partially partitioned across odorants evoking the two types of responses. The extent of this partitioning and the significance of it for coding will require more extensive analysis with more odorants than tested in the present study. It will be interesting to see if the extent of this partitioning is a biological variable across species that is selected for in evolution.

Depending on the extent of this partitioning it could have practical consequences for defining the molecular receptive range (MRR) of an ORN. The MRR is intended to represent the full set of odorants that interacts with a given receptor cell or its OR, and typically is determined by identifying odorants that evoke conventional excitatory (cyclic nucleotide-dependent) responses [36]. However, as we show here, odorants evoking inhibitory responses often have little or no inherent excitatory activity and would easily be missed in a conventional excitatory screen [14], [37]. In order to extend the definition of MRR to include odorants that activate PI3K-dependent (or any other type of) inhibitory signaling, it could be important to modify the protocol typically used to establish the MRR.

Each rodent ORN expresses only one of approximately 1,000 functional ORs [38], [39], [40], [41], suggesting that each response profile identified within a population of ORNs presumably represents the response of a different OR. Large scale profiling of conventional excitatory responses in mouse ORNs found 93 different response profiles in 217 odorant responsive cells [19]. In comparison, we found that 42 odorants evoked 20 different PI3K-dependent profiles in 51 ORNs, while 8 tomato derived odorants evoked 10 different PI3K-depedent profiles in 36 ORNs. These results show that odorants evoking PI3K-dependent responses target a significant fraction of the ORs, although possibly a smaller fraction of the ORs than those evoking conventional excitatory responses. However, the number of PI3K-dependent profiles in the ORN population we tested could reflect incomplete PI3K blockade, resulting in an underestimation of the fraction of PI3K-dependent response profiles. If so, the proportion of PI3K-dependent response profiles in the population potentially approaches or equals that of conventional excitatory response profiles, which would argue that excitatory and inhibitory odorants target equal numbers of ORs.

Our data do not directly address whether excitatory and inhibitory odorants target the same OR. However, finding that the PI3K-dependent inhibitory responses and the conventional excitatory responses of individual ORNs are tuned to different numbers of odorants and not the same odorants bears on the question. Given that each rodent ORN expresses only one of approximately 1,000 functional ORs [38], [39], [40], [41], and that PI3K-dependent odorants can activate cyclic-nucleotide signaling in mammalian ORNs when PI3K is blocked [14], it is reasonable to assume that PI3K-dependent odorants activate the canonical OR. If so, our finding that the PI3K-dependent inhibitory responses and the conventional excitatory responses of individual ORNs are tuned to different numbers of different odorants is consistent with the hypothesis that odorants evoking the two types of responses bind to different sites on the OR. Further work is required to address this important question.

Overall, finding that a large repertoire of different molecular classes of odorants, including those found in a behaviorally-salient complex odor mixture, can activate PI3K, and do so in a physiologically relevant context to olfactory transduction, argues that PI3K-dependent inhibition can provide a broad basis for opponent coding in mammalian olfaction. The extent to which inhibitory input contributes to the combinatorial code that is generally assumed to be the basis for odor coding and discrimination in many different animals [6], [8], [42], [43] remains to be determined. Finding that PI3K-dependent inhibition can provide a broad basis for opponent coding in mammalian olfaction underscores the importance of better understanding the extent and mechanisms through which inhibitory input in general contributes to the olfactory code.
